# Integrated multi-omic data reveal the potential molecular mechanisms of the nutrition and flavor in Liancheng white duck meat

**DOI:** 10.3389/fgene.2022.939585

**Published:** 2022-08-15

**Authors:** Hao Zhou, Yu Yang, Lixia Wang, Shengqiang Ye, Jiajia Liu, Ping Gong, Yunguo Qian, Huijun Zeng, Xing Chen

**Affiliations:** ^1^ Insitute of Animal Husbandry and Veterinary, Wuhan Academy of Agricultural Science, Wuhan, China; ^2^ Department of Animal Science, School of Agriculture and Biology, Shanghai Jiao Tong University, Shanghai, China; ^3^ Wuhan Institute for Food and Cosmetic Control, Wuhan, China; ^4^ Key Laboratory of Edible Oil Quality and Safety for State Market Regulation, Wuhan, China

**Keywords:** liancheng white ducks, multi-omics, umami-tasting compounds, poultry meat, meat nutrition

## Abstract

The Liancheng white (LW) duck is one of the most valued Chinese indigenous poultry breeds. Its meat is rich in nutrients and has distinct flavors, but the molecular mechanisms behind them are unknown. To address this issue, we measured and compared multi-omic data (genome, transcriptome, and metabolome) of breast meat from LW ducks and the Mianyang Shelduck (MS) ducks. We found that the LW duck has distinct breed-specific genetic features, including numerous mutant genes with differential expressions associated with amino acid metabolism and transport activities. The metabolome driven by genetic materials was also seen to differ between the two breeds. For example, several amino acids that are beneficial for human health, such as L-Arginine, L-Ornithine, and L-lysine, were found in considerably higher concentrations in LW muscle than in MS duck muscle (*p* < 0.05). *SLC7A6*, a mutant gene, was substantially upregulated in the LW group (*p* < 0.05), which may lead to excessive L-arginine and L-ornithine accumulation in LW duck meat through transport regulation. Further, guanosine monophosphate (GMP), an umami-tasting molecule, was considerably higher in LW muscle (*p* < 0.05), while L-Aspartic acid was significantly abundant in MS duck meat (*p* < 0.05), showing that the LW duck has a different umami formation. Overall, this study contributed to our understanding of the molecular mechanisms driving the enriched nutrients and distinct umami of LW duck meat, which will provide a useful reference for duck breeding.

## 1 Introduction

The Liancheng white duck (LW, *Anas platyrhynchos* domesticus), also called the “White Dove Duck,” is one of the precious and rare waterfowl resources in China. Historical sources note that LW ducks have been raised in Lian city for over 700 years. Because of the long process of artificial and natural selection, LW ducks have genetic stability and have evolved various characteristics, such as white feathers, black beaks, and black feet. Its meat is considered to be enriched in nutrients and has unique flavor (Bai et al., 2021; Zhang et al., 2021b; Huo et al., 2021). However, the underlying molecular mechanism remains unknown.

Recently, multi-omic association analysis has become popular in the investigation of potential molecular mechanisms in complex trait and disease studies (Theron et al., 2013; Douglas et al., 2018; Chen et al., 2020; Arora et al., 2021; Cardoso et al., 2021; Doran et al., 2021; Lu et al., 2021). It has been proven a powerful tool for studying muscle regulation systems that drive meat quality traits (Ye et al., 2017; Wang et al., 2020; Dou et al., 2022). Dou et al. (2022) used transcriptome and metabolome data to show that considerably high levels of muscle-useful metabolites, such as 13-HODE, NMN, and LPL, in black-boned chickens revealed essential health and therapeutic benefits. The integration of transcriptome and metabolome analysis in Bamei pork and Gansu Black pork also found essential genes and fatty acids that contribute to pork quality (Wang et al., 2020). Pork quality is also markedly related to vast quantitative trait loci (QTL) on the pig genome in the pig QTL database (Zhan et al., 2022), suggesting that the genetic factors play an important role in defining the characteristics of meat quality (Ye et al., 2017). However, the lack of genomic, transcriptomic, and metabolomic resources has limited the investigation into the molecular mechanisms of the meat characteristics of the LW duck.

In this study, we performed genome, transcriptome, and metabolome analyses to understand better the molecular mechanisms of the nutrients and meat flavor in LW duck meat. Another common indigenous shelduck, Mianyang Shelduck (MS, *A. platyrhynchos* domesticus) that has the same matrilineal origin from mallard (*A. platyrhynchos*) with LW duck (Li et al., 2010), was also measured using multi-omics and used as a control. Genetic variations, gene expressions, and metabolite contents in the breast meat of LW ducks were compared to those in MS duck meat. The biological gene–metabolite regulation was investigated in the breast meat of LW ducks. Overall, this study adds to our understanding of the molecular mechanisms behind the nutrition usage and meat flavor of the LW duck and will provide reference for duck breeding.

## 2 Materials and methods

### 2.1 Sample collection

LW ducks and MS ducks were reared to 300 days of age on the same farm, under the same standard management conditions. They were kept together from duckling to adulthood, fed a commercial corn–soybean-based diet *ad libitum* with free access to water and fasted for 8 h before being slaughtered. All ducks were healthy and had not been treated with antibiotics. In total, 16 female ducks (eight from each group) were randomly selected and fasted for 6 h. We took breast muscle samples and immediately stored them in liquid nitrogen for the following experiments.

### 2.2 Genome sequencing and analysis

Tissue from sixteen breast muscles (eight from each breed) was sampled for DNA extraction. DN easy tissue extraction kits (Qiagen, Valencia, CA, United States) were used to extract genomic DNA following the manufacturer’s protocol. Next-generation sequencing (NGS) technology was used to perform paired-end (PE) sequencing of the libraries based on the Illumina sequencing platform. The sequencing depth was 40 × for each sample. Reads were filtered and aligned to the reference genome of *A. platyrhynchos* domesticus (CAUduck1.0, http://uswest.ensembl.org/Anas_platyrhynchos_platyrhynchos/Info/Index) using the bwa software (Li and Durbin, 2009). Duplicate reads were removed from individual alignments using the Picard MarkDuplicates tool (v1.115, https://github.com/broadinstitute/picard). The Genome Analysis Toolkit (GATK) Haplotype Caller protocol was used to call single-nucleotide polymorphisms (SNPs) and small insertions/deletions (INDELs) via the local re-assembly of haplotypes (Mckenna et al., 2010). The SNPs and INDELs were filtered prior to analysis with the GATK Variant Filtration protocol (Mckenna et al., 2010). The filter settings were as follows: QD < 10.0, ReadPos RankSum < − 8.0, FS > 10, QUAL <30, DP < 4. Then, Fst values of single SNPs and INDELs were calculated using vcftools v.0.1.13 (Danecek et al., 2011) to evaluate genetic differentiation. The range of FST is between 0 and 1. An FST value of 0 indicated that the mutations have no genetic divergence between populations and a value of 1 indicated the mutations have complete extreme division (Pino Del Carpio et al., 2011). SNPs and INDELs with the top 1% Fst values were taken to indicate selected mutations, which referred to previous comparative genomic studies (Islam et al., 2019; Liu et al., 2019; Xiong et al., 2019). They then were annotated by the SnpEff tool, which offered putative variant impacts (high, moderate, low, modifier) to categorize and prioritize variants on genes (Cingolani et al., 2012). A functional enrichment analysis of genes with mutations was performed using the Functional Annotation Chart tool in Database for Annotation, Visualization and Integrated Discovery (DAVID) (https://david-d.ncifcrf.gov/), using duck as background. *p* < 0.05 was set as the threshold for significantly enriched GO terms.

### 2.3 RNA-seq and analysis

Six breast muscle samples (three from each breed) were collected at random. We used the Trizol protocol for total RNA extraction of the samples. RNA sequencing was carried out on the Illumina HiSeq platform using protocols for 2 × 150 bp paired-end sequencing with the aim of obtaining 6G raw data from mRNA. After sequencing, raw data were trimmed and filtered using Trimmomatic 0.33 (Bolger et al., 2014). We followed the HISAT2, StringTie, and R package Ballgown protocol for gene expression quantification (Kim et al., 2015; Pertea et al., 2016). Paired-end reads were aligned to the reference sequence of *A. platyrhynchos* domesticus (CAUduck1.0) using HISAT2, and BAM files were sorted and indexed by SAMtools (v.1.3) (Li et al., 2009). Then, StringTie (v.1.3.1) was used to assemble transcripts, estimate transcript abundances, and create table counts for each sample. Finally, Ballgown (v.2.8.4) was used to extract gene-level expression measurements and call DEGs (|Log2FoldChange|≥0.5, *p*-value <0.05) from the Ballgown objects obtained from StringTie. The statistical test in ballgown is a parametric F-test comparing nested linear models (Frazee et al., 2015). A functional enrichment analysis of DEGs was performed using the Functional Annotation Chart tool in DAVID (https://david-d.ncifcrf.gov/), using duck as background. *p* < 0.05 was set as the threshold for screening significantly enriched GO terms. The DEGs were overlapped with the mutated genes, these overlapped genes were input to the Functional Annotation Table tool in DAVID for functional analysis.

### 2.4 Metabolite-seq and analysis

Metabolites in the breast muscle tissue of twelve ducks (six from each breed) were quantitatively profiled using an LC-MS/MS-based targeted metabolomics platform, as described previously (Bao et al., 2019). In brief, metabolites in muscle homogenates were extracted with methanol. The samples were incubated on ice for 5 min and then were centrifuged at 15,000 g, 4°C for 20 min. Some of the supernatant was diluted to a final concentration containing 53% methanol by LC-MS grade water. The samples were subsequently transferred to a fresh Eppendorf tube and then were centrifuged at 15,000 g, 4°C for 20 min. Finally, the 2 μL supernatant was injected into a SCIEX QTRAP^®^ 6,500 + system (SCIEX, Framingham, MA, United States). The eluents were eluent A (0.1% Formic acid-water) and eluent B (0.1%Formic acid-acetonitrile). The solvent gradient was set as follows: 2% B, 2 min; 2–100% B, 15.0 min; 100% B, 17.0 min; 100–2% B, 17.1 min; 2% B, 20 min.

Metabolomics data analyses were carried out using the SCIEX OS 1.4 software (SCIEX, Framingham, United States), which performed the integration and correction of chromatographic peaks. The peak area of each chromatogram peak reflects the relative content of the related metabolites, and all the chromatographic peak area-integral data are finally saved. We corrected the detected mass spectrum peaks of each metabolite in the samples to ensure the accuracy of the qualitative and quantitative analyses through the quality control sample. An in-house novoDB (novogene database) was used to identify metabolites. The variable importance in the projection (VIP) score is used to estimate the important metabolites in a PLS-DA model. These metabolites were subsequently assessed using Student’s *t* test. Significantly differential metabolites (SDMs) were identified according to the following criteria: VIP value >1, fold change > 1.2 or fold change < 0.833, and *p*-value < 0.05. SDMs were then used for the KEGG pathway enrichment analysis, with significant criteria considered when *p* < 0.05. Metabolite–metabolite correlations were analyzed using Pearson’s correlation in the R software. Statistical significance was determined using the correlation *t*-test with *p* < 0.05. The metabolite correlation heatmap was drawn based on the correlation coefficients.

### 2.5 Joint analysis of transcriptomic and metabolomic data

To investigate the potential regulatory network of SDMs and DEGs, the expression profile matrix of DEGs and corresponding metabolic profile matrix of SDMs of six individuals (three from each breed) were prepared for correlation analysis. Correlation analyses were carried out using the Pearson method provided by R packages psych (v.1.8.12, https://CRAN.R-project.org/package=psych). Test analyses for correlation coefficients were performed by corr. test using the FDR method for multiple tests. A strong correlation between DEGs and SDMs was identified with the threshold |r|>0.8 and corrected *p* < 0.05, and then the significantly correlated combinations were selected and used to construct the network.

## 3 Results

### 3.1 Genetic characteristics of liancheng white duck

We performed whole-genome resequencing of 16 samples from LW ducks and MS ducks. In total, 635 Gb of raw reads was generated, with an average depth of 40 × (Supplementary Table S1A). These data were mapped against the duck reference assembly and called variants. In total, 4,008,770 INDELs and 12,332,104 SNPs were identified and used for the subsequent analysis.

**FIGURE 1:**
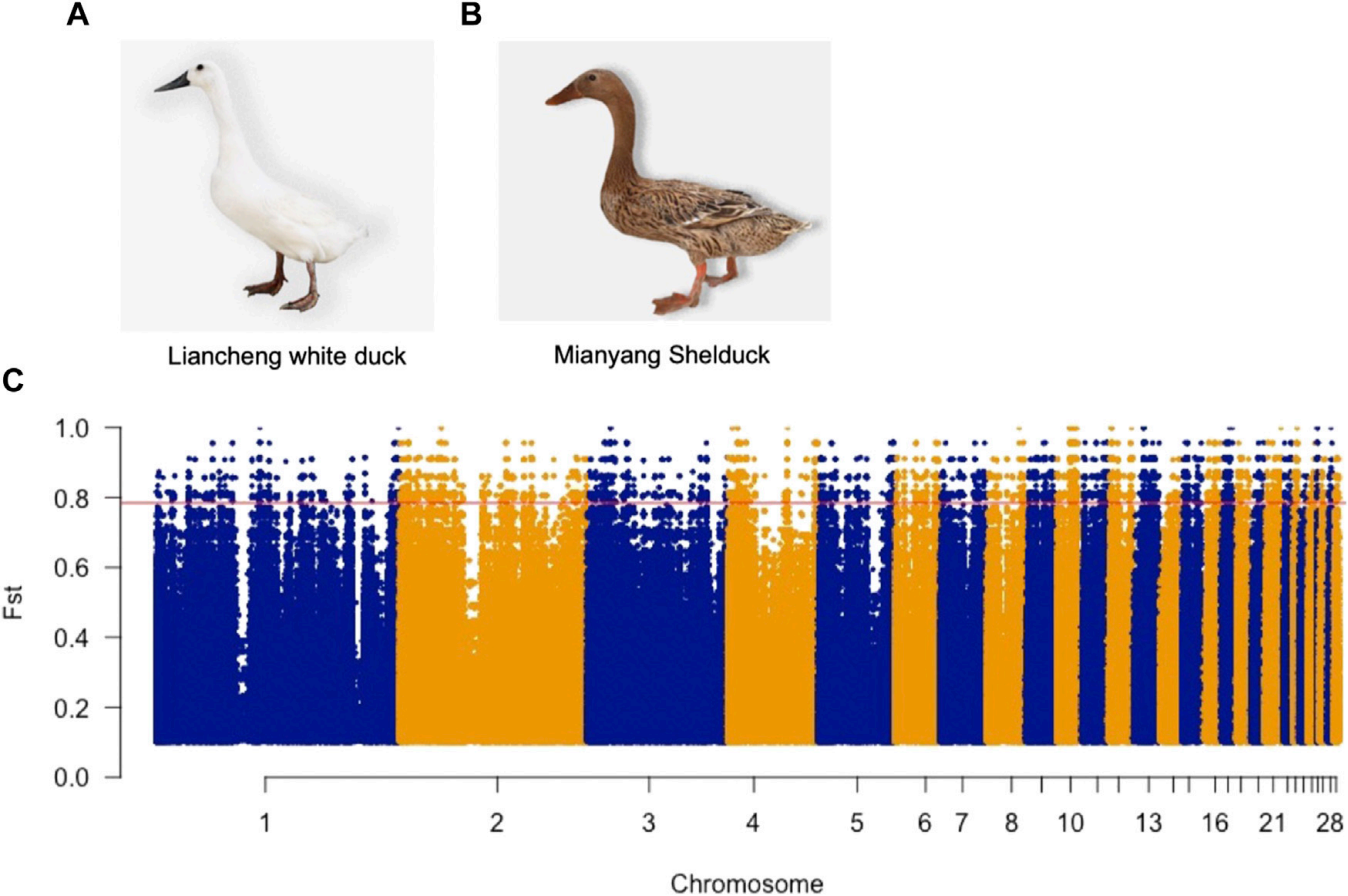
Photograph of the Liancheng white duck **(A)** and Mianyang shelduck **(B)**. **(C)** Manhattan plots illustrate the Fst value between Liancheng white duck and Mianyang Shelduck. The red line represents the Top 1% Fst value threshold (Fst >0.78).

To determine the genetic features of LW ducks, we explored the genomic landscape of SNP Fst values to identify candidate genes that potentially control specific traits present in two breeds. We identified 133,448 SNPs and INDELs with Fst values that were in the top 1% (Fst >0.78) (Figure 1) and thus were potentially positively selected in LW ducks (Supplementary Table S2A). Annotation of these SNPs indicated them to be located in 4,891 genes (Supplementary Table S2B). Further, 120 high effect mutations and 1,212 moderate effect mutations were located in 65 genes and 368 genes, respectively (Supplementary Table S2B). Indeed, several candidate genes with genetic variations have been shown to be associated with functions related to the meat traits of LW ducks (Supplementary Table S2C). One high effect mutation caused a frameshift in the sphingosine-1-phosphate lyase 1 (*SGPL1*) gene that was involved in the fatty acid metabolic process. We identified 12 mutations, including one nonsynonymous and eight synonymous, and three splice region variants on *TMLHE* involved in Lysine degradation and Trimethyllysine dioxygenase activity. In addition, one missense variant and 10 downstream gene variants were identified in *GLUL* that play roles in arginine biosynthesis and aspartate and glutamate metabolism. *SLC7A6* associated with Arginine transport has one missense variant and one upstream gene variant. *PLA2G6* and *ARRDC3*, fat and lipid metabolism-related genes, have 13 and 45 modifier effect variants, respectively, which may affect the corresponding gene expression. Further, several intron variants were found in *SIRT7* and *NAMPT*. Overall, these gene variants may be responsible for the meat characteristics of LW ducks.

The 390 genes with high and moderate effect mutations are functionally enriched in several related metabolic processes, such as the beta-alanine biosynthetic process, lipid metabolic process, catabolic process, and galactose catabolic process via UDP-galactose (Supplementary Table S2C). The *PLCXD3, PLA2G6, PLCH1, PAFAH2, GAL3ST2, DPYD,* and *GALT* genes were observed to be involved in most of these functions.

### 3.2 Gene expression profiles of breast meat differ between liancheng white ducks and mianyang shelduck

Transcriptome sequencing was used to detect the mRNA expression profiles of the breast muscles of LW ducks and MS ducks. In total, approximately 254 million clean reads were obtained and about Q30 of the sequence reads per sample were about 95, suggesting a good sequence quality (Supplementary Table S1B).

**FIGURE 2:**
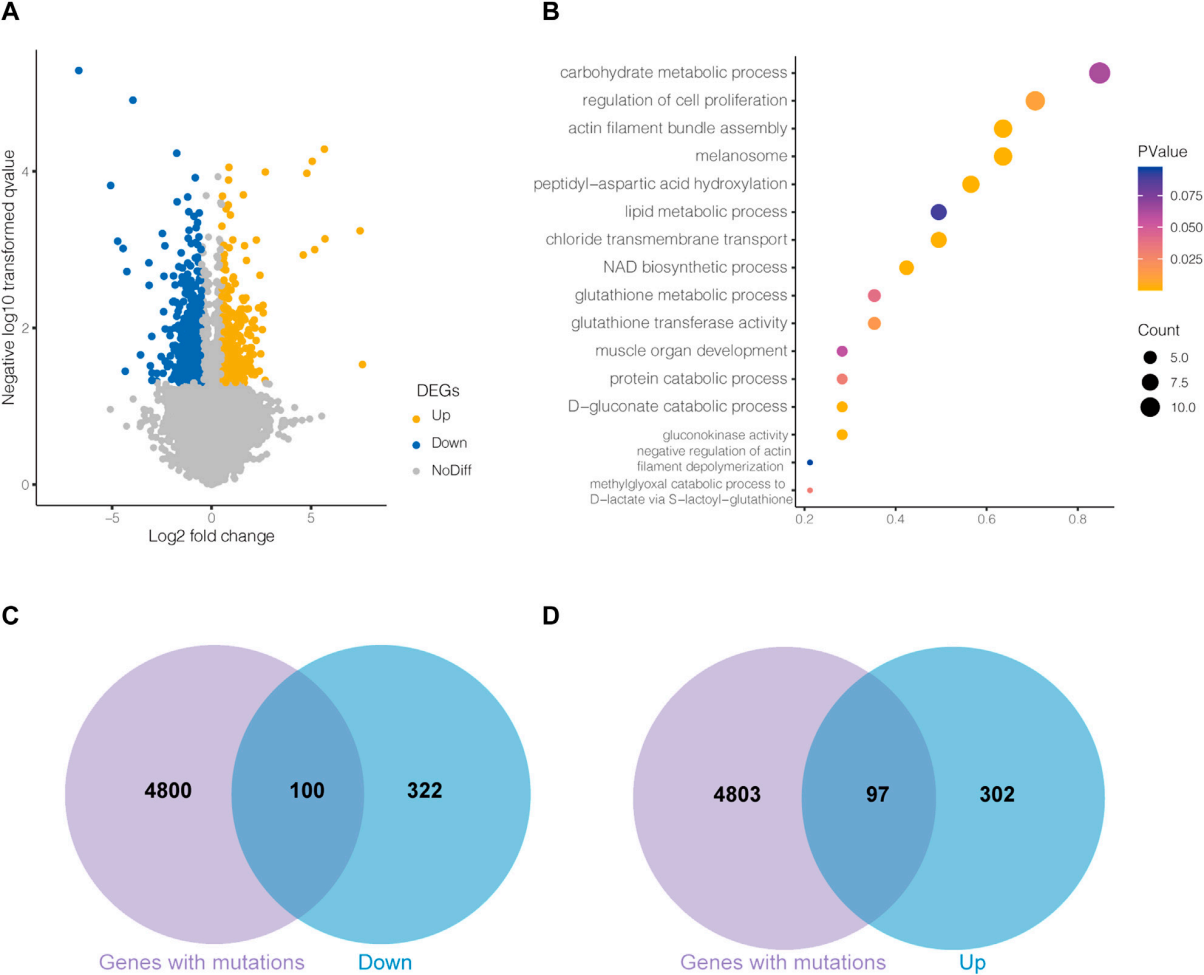
**(A)**Volcano plot of differentially expressed genes (DEGs) between Liancheng white (LW) duck and Mianyang Shelduck. **(B)** Bubble plot for visualizing several GO annotations of DEGs. The horizontal axis refers to gene ratio (count of input genes/count of genes in the GO term). The Venn plots show the overlaps between downregulated DEGs in LW duck and mutated genes **(C)** and upregulated DEGs in LW duck and mutated genes **(D)**.

The transcriptome differences were analyzed by comparing LW ducks and MS ducks. The comparison revealed 859 significantly differentially expressed genes (DEGs), including 415 upregulated and 444 downregulated genes in the LW ducks (*p* < 0.05). Of them, *ARRDC3,CASTOR2,CTH*, G*LUL,SLC7A6,* and *SIRT7* were significantly upregulated in LW duck compared to MS ducks, while *NAMPT, CHRND,* and *PDGFRB* were significantly upregulated in MS duck compared to LW ducks. The details of all DEGs found in the compared breeds are shown in Supplementary Table S3A, and DEGs are shown on the volcano plot (Figure 2A).

The DAVID website was used to identify the DEG functions. Overall, 81 significantly enriched entries were identified in the biological process category (*p* < 0.05, Supplementary Table S3B). Most biological process-enriched items in the comparison were significantly associated with peptidyl-aspartic acid hydroxylation, the D-gluconate catabolic process, the methylglyoxal catabolic process to D-lactate via S-lactoyl-glutathione, the glutathione metabolic process, and the lipid metabolic process (Figure 2B). In molecule-enriched functions, gluconokinase activity and glutathione transferase activity were also enriched.

### 3.3 Integrative analysis of the genome and transcriptome

To investigate the DEGs genetically regulated, one hundred downregulated DEGs and ninety-eight upregulated DEGs in LW duck were identified and may be regulated by genetic mutations (Figures 2C,D). Among them, we investigated 9 upregulated DEGs in LW duck with genomic mutations that are related to amino acid metabolic and lipid-related functions (Table 1), which are strongly associated with the formation and quality of meat, as a previous report demonstrated (Zhan et al., 2022). Several significantly upregulated genes in the LW group, such as *CTH, CASTOR2, GLUL, SIRT7*, and *SLC7A6*, participate in amino acid metabolism and transport. *GLUL* also play roles in aspartate and glutamate metabolism. *ARRDC3, PLA2G6,* and *SGPL1,* three important genes controlling lipid and fatty acid metabolic processes, were observed to be significantly upregulated expressed (*p* < 0.05). The NAMPT expression was significantly upregulated in the MS group when compared to LW duck (*p* < 0.05), which was related to functions of nicotinamide metabolism.

**TABLE 1:** The upregulated differentially expressed genes with genetic variations in LW duck shown to have functions involved in amino acid metabolic and lipid-related functions (*p* < 0.05).

ID	log2FoldChange	*p*-value	High and moderate effect variants^a^	Low and modifier effect variants^b^	Related functions^c^
ARRDC3	1.21	0.05	0	45	Fat pad development (GO: 0060613)
CASTOR2	1.59	0.03	0	216	Cellular response to amino acid starvation (GO:0034198)
CTH	1.01	0.04	0	1	lipid metabolic process (GO: 0006629), cysteine metabolic process (GO: 0006534)
GLUL	1.12	0.02	1	10	glutamine family amino acid metabolic process (GO: 0009064), glutamine catabolic process (GO: 0006542)
PLA2G6	2.01	0.02	0	13	Lipid metabolic process (GO:0006629), Linoleic acid metabolism (GO: 0032049)
PLIN2	1.6	0.02	1	20	Lipid storage (GO: 0019915)
SGPL1	0.8	0.02	1	96	Fatty acid metabolic process (GO: 0006631)
SIRT7	0.6	0.04	0	4	peptidyl-lysine deglutarylation (GO: 0061699)
SLC7A6	0.78	0.01	1	1	Ornithine transport, amino acid transport (GO: 0003333)

a The number of high and moderate effect variants.

b The number of low and modifier effect variants.

c Related Functions: The genes functions were obtained using DAVID Functional Annotation Tools (http://david.abcc.ncifcrf.gov/).

### 3.4 Metabolic profiles of breast meat differ between liancheng white duck and mianyang shelduck duck

**FIGURE 3:**
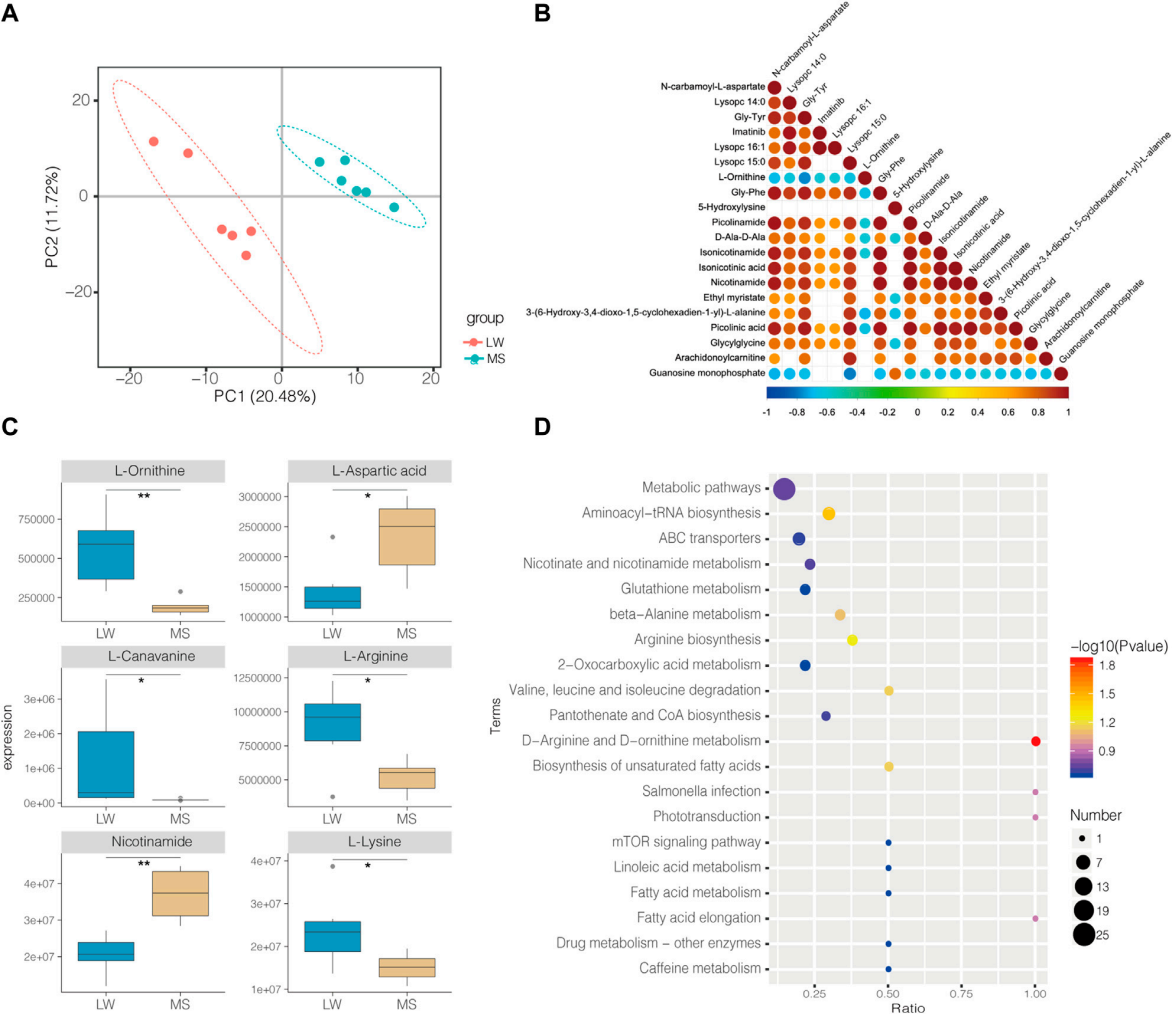
**(A)** PLS-DA Score Scatter plot of metabolites in Liancheng white duck and Mianyang Shelduck. **(B)** Heatmaps showing correlation patterns of top 20 significantly differential metabolites (SDMs) between LW duck and MS duck. **(C)** Box plot showing six SDMs. *Indicate *p*-value < 0.05; **indicate *p* value < 0.01. **(D)** Bubble plot for visualizing GO Annotation of SDMs.

In total, 585 metabolites were identified. Among them, Creatine, D-Glucose 6-phosphate, D-Mannose 6-phosphate, Taurine, and Anserine are the top five abundant metabolites, supporting results from other studies on ducks (Liu et al., 2013; Wang et al., 2020). PLS-DA analysis demonstrates that the samples from different groups were separated and classified into two distinct clusters (Figure 3A). The first PLS component (PC 1) accounted for 20.5% of the whole variation (Figure 3A). Screening yielded 83 significantly differential metabolites (SDMs), comprised of 16 and 64 metabolites upregulated and downregulated in the muscles of LW duck when compared to MS duck, respectively (VIP value > 1, fold change>1.2 or fold change< 0.833, and *p* < 0.05, Supplementary Table S4). L-Arginine, L-Ornithine, Trimethyllysine, L-Lysine, Guanosine monophosphate (GMP), and Xanthosine were significantly upregulated in the LW group when compared to MS duck (Figure 3C). Of them, L-Ornithine was the most significantly upregulated metabolite (Fold change = 2.92, *p* = 0.001) (Table 2). Whiles L-Aspartic acid and Nicotinamide were significantly upregulated in MS ducks when compared to LW duck (Figure 3C). Nicotinamide metabolism was proven regulated by the *NAMPT* gene (Zhan et al., 2022), which was significantly upregulated in the MS group compared to LW group and was reflected by the significantly changed compound nicotinamide in MS duck meat. In addition, most of SDMs exhibited a high correlation with other different metabolites, and most SDMs were negatively associated with L-Ornithine and Guanosine monophosphate (Figure 3B).

**TABLE 2:** The upregulated significantly differential metabolites (SDMs) in LW duck compared to MS duck (VIP value > 1, fold change>1.2 or fold change< 0.833, and *p* < 0.05).

SDMs	Formula	Molecular weight	Class	Fold Change	*P*	VIP
L-Ornithine	C5H12N2O2	132.16	Amino Acid And Its Derivatives	2.92	0.001	2.02
5-Hydroxylysine	C6H15ClN2O3	198.65	Amino Acid And Its Derivatives	2.06	0.002	1.42
Trimethyllysine	C9H21N2O2	189.275	Amino Acid And Its Derivatives	1.55	0.033	1.2
L-Lysine	C6H14N2O2	146.19	Amino Acid And Its Derivatives	1.58	0.037	1.39
L-Arginine	C6H14N4O2	174.2	Amino Acid And Its Derivatives	1.7	0.041	1.29
L-Canavanine	C5H12N4O3	176.174	Amino Acid And Its Derivatives	12.41	0.042	1.88
Indole-3-acetamide	C10H10N2O	174.2	Indole And Its Derivatives	1.69	0.017	1.39
Serotonin hydrochloride	C10H13ClN2O	212.677	Indole And Its Derivatives	12.41	0.046	1.82
Guanosine monophosphate	C10H14N5O8P	363.058	Nucleotide And Its Derivates	2.77	0.005	1.65
Deoxyribose 5-Phosphate	C5H11O7P	214.11	Nucleotide And Its Derivates	1.48	0.009	1.97
Xanthosine	C10H12N4O6	284.225	Nucleotide And Its Derivates	1.84	0.024	1.7
CDP	C9H15N3O11P2	403.176	Nucleotide And Its Derivates	3.41	0.026	1.54
Isobutyrylglycine	C6H11NO3	145.156	Organic Acid And Its Derivatives	1.48	0.013	1.76
Homotaurine	C3H9NO3S	139.17	Organic Acid And Its Derivatives	1.74	0.024	1.35
Allantoin	C4H6N4O3	158.12	Organoheterocyclic compounds	1.92	0.005	1.8
Trimethylamine N-oxide	C3H9NO	75.11	Polyamine	2.57	0.014	1.4

By annotating their functions and clustering the differential metabolites based on KEGG analysis, the results indicate that SDMs were significantly enriched in 40 pathways (Supplementary Table S4B, Figure 3D). Among them, “D-Arginine and D-ornithine metabolism” (map00472), and “Aminoacyl-tRNA biosynthesis” (map00970) was significantly enriched (*p* < 0.05). The levels of six differential metabolites in these pathways differed significantly (*p* < 0.05), including L-Ornithine, L-Arginine, L-Leucine, L-Lysine, L-Aspartic acid, and L-Cysteine.

### 3.5 Interactive network analysis of metabolomes and transcriptomes

**FIGURE 4:**
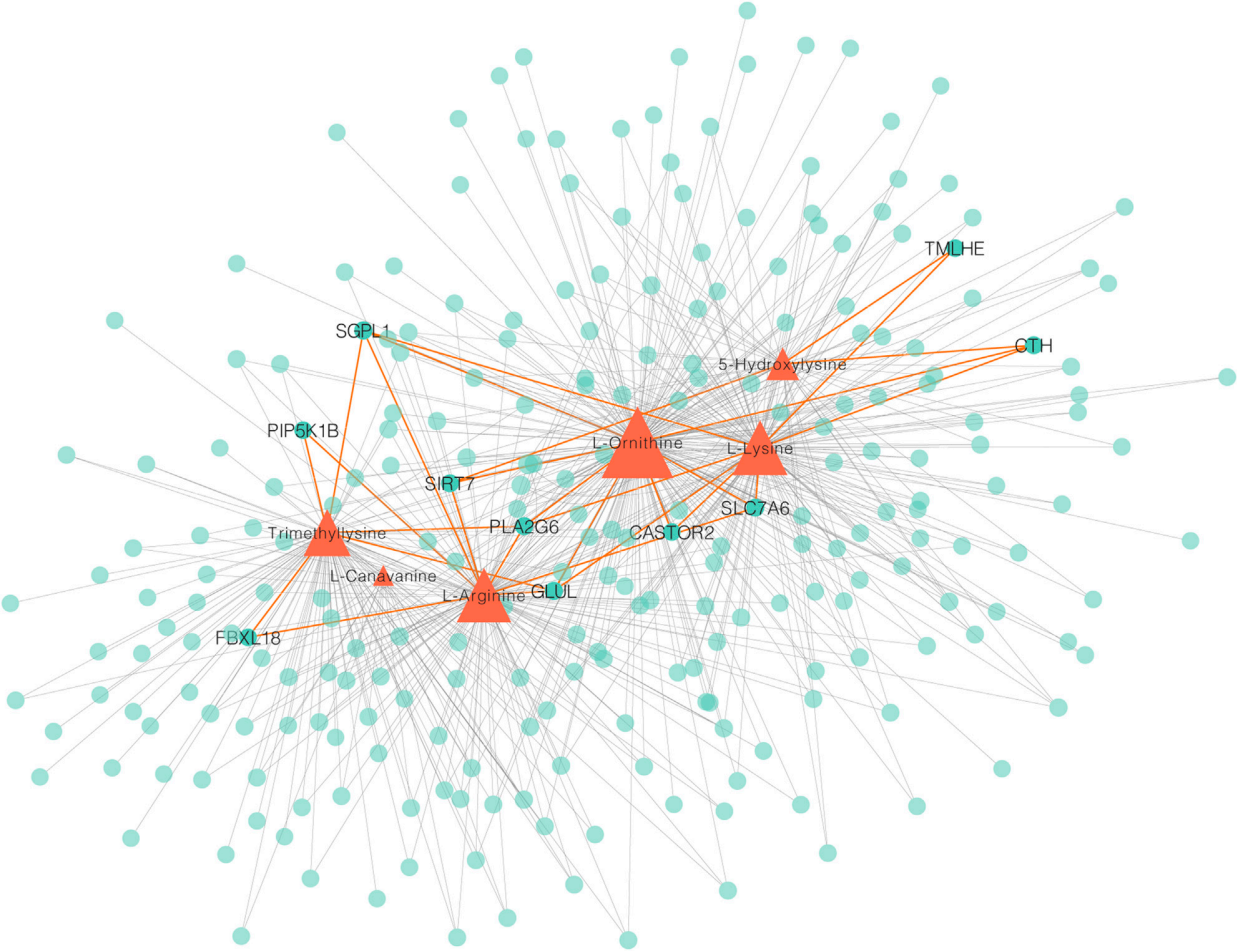
A Network integrating gene–metabolite associations. Orange triangles indicate metabolites, green circles indicate genes. Important connects are highlighted in orange.

To measure the associations between metabolomes and transcriptomes in the breast muscle of LW ducks and MS ducks, 859 DEGs and 83 SDMs were analyzed together in a correlation analysis. The focus on a strong correction showed 702 and 2,857 connections between DEGs and upregulated metabolites in LW ducks and MS ducks, respectively (r > 0.8, corrected *p* < 0.01, Pearson method) (Supplementary Table S5). The network of DEGs and upregulated metabolites in LW ducks was focused, and the results indicated that L-Ornithine was a key metabolite significantly positively correlated with the expression of multiple genes (Figure 4; Supplementary Table S5), including *SLC7A6* (*r* = 0.98, corrected *p* = 0.0006), *SIRT7* (*r* = 0.91, corrected *p* = 0.01)*, GLUL* (*r* = 0.96, corrected *p* = 0.003)*, CTH* (*r* = 0.91, corrected *p* = 0.01), *PLA2G6* (*r* = 0.95, corrected *p* = 0.003), and *CASTOR2* (*r* = 0.82, corrected *p* = 0.05). L-Arginine also was positively connected to the *SLC7A6* (*r* = 0.88, corrected *p* = 0.02)*, SIRT7* (*r* = 0.87, corrected *p* = 0.02), and *GLUL* (*r* = 0.96, corrected *p* = 0.002). In addition, L-Lysine positively correlated with the *TMLHE* expression (*r* = 0.87, corrected *p* = 0.02). These correlations suggested the possible regulation relationship between these genes and metabolites.

## 4 Discussion

The LW duck experienced a long-term domestication process, which resulted in the peculiar breed genetic traits discovered in this study, including many mutant genes with differential expressions involved in amino acid and fatty acid metabolism or transport activities. For example, one high effect mutation led to a frameshift in the *SGPL1* gene, which was involved in the fatty acid metabolic process that was previously reported (Bektas et al., 2010). The metabolome data, potentially driven by genetic material, were also discovered to differ from LW ducks to MS ducks, which is consistent with the finding that the composition of free amino acids in animal meat, which contributes to meat scent and flavor, differs among breeds (Dou et al., 2022; Zhan et al., 2022). All these findings may be the root cause of the exceptional taste of LW duck meat.

Several amino acids that are beneficial for human health, such as L-Arginine, L-Ornithine, and L-Lysine, showed significantly higher levels in LW breast meat compared to MS breast meat. L-arginine is an essential amino acid in the fetus and neonate, and it is conditionally an essential nutrient for adults, particularly in certain disease conditions. L-arginine administration is beneficial in improving reproductive, cardiovascular (Popolo et al., 2014), pulmonary (Mitani et al., 1997), renal (Ketteler et al., 1994), gastrointestinal (Lojo et al., 2016), liver (Lojo et al., 2016), and immune functions (Geiger et al., 2016), as well as in facilitating wound healing (Wu et al., 2000). In muscle cells, L-arginine can be hydrolyzed to L-ornithine, which exerts anabolic or anticatabolic effects through their involvement in protein metabolism, immune response, and cell proliferation (De Bandt and Cynober, 1998; Durante et al., 2007). Further investigation of the underlying mechanism in the present study found that because the content of metabolites, including L-arginine and L-ornithine, was upregulated, the expression level of *SLC7A6* was increased. *SLC7A6* is an important subunit of the transport system y + L for L-arginine transport (Rotmann et al., 2007; Barilli et al., 2014). A high correction between the *SLC7A6* gene and L-arginine and L-ornithine further confirms the regulation relationship between *SLC7A6* and these metabolites. A mutation is present in the five upstream regions of the *SLC7A6* gene and hence could cause the altered expression levels in LW meat. Taken together, these findings elucidate the roles and underlying mechanisms of *SLC7A6* in the genetic regulation of L-arginine and L-ornithine, which may contribute to high L-arginine and L-ornithine accumulation in the muscle of LW ducks. L-canavanine, a structural analogue of L-arginine (Rosenthal, 1977), was also highly accumulated in the muscle of LW ducks. L-canavanine showed demonstrative antineoplastic activity against many human cancers (Ding et al., 1999; Nurcahyanti and Wink, 2016), increasing the nutrition of LW duck meat.

L-lysine is an amino acid required by animals. It is known as the first limiting amino acid, and it plays a vital role in human physiologic development and fatty acid oxidation (Jin et al., 2020; Matthews, 2020). Although lysine is a common amino acid in body proteins, it is scarce in many key dietary sources (e.g., grains or meat of animals fed an exclusively grain-based diet) (Matthews, 2020). In the current study, a greater L-lysine level was discovered in the meat of LW ducks. Fürst (1993) discovered that adding L-lysine to one’s diet might help cure osteoporosis, while Shimomura et al. (2014) found that modest dietary supplementation with L-lysine might reduce vascular calcification in uremic rats. In the current work, the *TMLHE* gene, which encodes Trimethyllysine dioxygenase and is involved in the L-lysine degradation pathway, was shown to be significantly downregulated in LW ducks, and it might be responsible for the higher lysine content in LW duck meat. In addition, 12 affected variants in the *TMLHE* gene were found. These mutations, as intrinsic genetic factors, may be responsible for modifying *TMLHE* functions or expressions, further impacting L-lysine accumulation in the meat of LW ducks.

The breast meat of LW ducks and MS ducks is enriched differentially in Umami-tasting compounds. GMP was significantly upregulated in LW duck meat, and it is used widely as an umami taste stimulus and potent flavor enhancer, as it synergistically increases the umami taste elicited by glutamate, another important umami-tasting compound (Kuninaka, 1960; Ninomiya, 2002; Lin et al., 2003). In the present study, there is no difference in glutamate abundance between LW and MS ducks, suggesting GMP may be the major driver of umami taste in LW duck meat. However, another ubiquitous umami compound (Ney, 1971; Zhang et al., 2021a), L-Aspartic acid was higher abundant in the breast meat of MS ducks than that of LW duck, suggesting the different umami formations of LW duck meat. Aspartic Acid plays an important role in the synthesis of other amino acids and in the urea cycles. It is also a nitrogen donor for arginine synthesis (Reitzer, 2004). Lower abundant L-Aspartic acid in LW duck (higher abundant in MS duck) maybe due to consumption in the synthesis of highly abundant arginine in LW duck.

In this study, the regulation relationship of mutated DEGs and SDMs in LW duck meat was identified based on an integrative analysis of multi-omic data. For example, the mutated genes SLC7A6 and TMLHE, correlated with several beneficial metabolites, such as L-Arginine, L-Ornithine, and L-Lysine, may play roles in the high accumulation of these beneficial metabolites in LW duck meat. Differential content of umami metabolites, such as GMP and L-Aspartic acid in LW duck meat, may be an important factor in the formation of the unique flavor of LW duck meat. Overall, these results provide effective information and more evidence to support further insight into the biological mechanisms of LW duck meat.

## Data Availability

The multi-omics data analyzed during this study are available in the Sequence Read Archive (https://www.ncbi.nlm.nih.gov/sra) with the accession codes PRJNA864643.
